# Hydroethanolic Extract of *Salvia officinalis* L. Leaves Improves Memory and Alleviates Neuroinflammation in ICR Mice

**DOI:** 10.1155/tswj/2198542

**Published:** 2025-03-20

**Authors:** Bernard Sefah, Yolanda Ashie, Newman Osafo, Priscilla Kolibea Mante

**Affiliations:** ^1^Department of Pharmacology, Kwame Nkrumah University of Science and Technology, Kumasi, Ghana; ^2^Department of Pathology, Komfo Anokye Teaching Hospital, Kumasi, Ghana

**Keywords:** cognitive impairment, lipopolysaccharide, neuroinflammation, *Salvia officinalis*, TNF-*α*

## Abstract

Neurodegenerative disorders are known to be commonly associated with neuroinflammation. Plants with antioxidant and anti-inflammatory properties hold prospect in alleviating neuroinflammation. One such plant with documented anti-inflammatory and antioxidant potential is *Salvia officinalis* L. This study looked at effects of the hydroethanolic leaf extract of *S. officinalis* L. on lipopolysaccharide (LPS)-induced neuroinflammation and associated memory impairment using an ICR mouse model. Assessment of the phytochemical constituents in *S. officinalis* L. and its acute toxicity was conducted. Mice were treated with *S. officinalis* L. extract (30, 100, and 300 mg/kg) after LPS administration. Object recognition and elevated plus maze tests were employed to assess neuroinflammation-induced behavioral changes. Brain samples were taken to determine the levels of TNF-*α* and conduct histopathological analysis. The hydroethanolic extract of *S. officinalis* L. was found to contain alkaloids, glycoside, tannins, flavonoids, and coumarins and exhibited no observable acute toxicity. The extract showed the presence of eicosatrienoic acid, methyl ester, and phenanthrene derivatives. The extract improved memory and cognitive performance but had no significant effect on brain tissue TNF-*α* expression. *S. officinalis* L. treatment in mice with neuroinflammation also resulted in reduced mononuclear infiltration and gliosis and reduced apoptotic and necrotic neurons as well as no observable brain lesions. *S. officinalis* L. holds promising pharmacological activity at reducing neuroinflammation and its associated cognitive impairment.

## 1. Introduction

The effect of neuroinflammation on the increased risk of cognitive decline is becoming increasingly apparent in recent studies. It is commonly known that neurodegenerative diseases such as Alzheimer's disease (AD), Parkinson's disease (PD), and HIV-associated neurocognitive disorders (HANDs) are frequently associated with neuroinflammation [1–3]. However, defining neuroinflammation remains a challenge. DiSabato et al. [4] define neuroinflammation as an inflammatory response that takes place inside the brain or spinal cord. Many chemicals, including reactive oxygen species (ROS), chemokines, secondary messengers, and cytokines, are released, which set off this reaction. Endothelial cells originating from sources outside the central nervous system (CNS) and CNS glial cells—particularly astrocytes and microglia—produce these chemicals. Increases in ROS and cytokine production, such as tumor necrosis factor-*α* (TNF-*α*), together with other inflammatory mediators, are characteristics of chronic and uncontrolled inflammation. Microglia, which serve as the immune surveillance cells and perform macrophage-like functions in the CNS, perform an important role in the process of neuroinflammation [4].

Lipopolysaccharide (LPS)-induced neuroinflammation is a widely used model of neuroinflammation, which involves microglia activation in the brain [5]. LPS or endotoxin comes from the outer membrane of gram-negative bacteria [6]. LPS-induced neuroinflammation comprises the binding of LPS to CD14 on microglia membranes. This interaction triggers the activation of microglia through Toll-like receptor-4 (TLR-4) [6]. Microglia activation then initiates various signal transduction mechanisms, including mitogen-activated protein kinase (MAPK) and phosphoinositide-3 kinase/protein kinase B (PI3K/AKT) [7]. Ultimately, these processes lead to the activation of NF-*κ*B (nuclear factor kappa-light-chain-enhancer of activated B cells). Consequently, NF-*κ*B activation results in the synthesis of cyclooxygenase-2 (COX-2), inducible nitric oxide synthase (iNOS), cytokines, and chemokines [7].

*Salvia officinalis* L., also known as sage, is a perennial rounded shrub within the family Lamiaceae. It is the most extensive genus within the Lamiaceae family, comprising nearly 900 species. The name *Salvia* originated from a word in Latin which means “to heal,” perfectly capturing the legendary notion that it has “magical” healing powers for a variety of diseases, hence its widespread use in traditional medicine [8]. The species *S. officinalis* L.'s native root is traced to the Mediterranean and Middle Eastern regions, but it has become naturalized in various countries across the globe [9]. In Africa, *S. officinalis* L. holds significant importance in traditional medicine. *Salvia* is widely employed for the treatment of many kinds of illnesses which include inflammation, memory loss, cancer, malaria, and microbial infections, as well as the disinfection of dwellings following illness [10]. It is utilized by different African tribes for a wide range of illnesses, including ethnoveterinary uses and as disinfectants [11]. It is one among the spices and herbs that are cultivated in Kenya for culinary purposes, and its significance is growing [11].

Sage is renowned for its robust antioxidant and anti-inflammatory characteristics. An ethanol extract of sage leaf was observed to effectively suppress the manufacture of interleukin-6 (IL-6), nitric oxide (NO), and TNF-*α* associated with RAW 264.7 macrophages that were stimulated by LPS [12]. Also, the administration of sage extract orally to mice before an LPS injection resulted in the inhibition of NO production and liver iNOS expression [13]. Other studies have also demonstrated *S. officinalis* L.'s neuroprotective effects. Sage aqueous extract protected rat cerebellar granule cells from amyloid beta (A*β*)–induced neurotoxicity by reducing apoptosis and oxidative damage [14]. At micromolar concentrations, carnosic acid and carnosol isolated from sage leaf extracts inhibited acetylcholinesterase (AChE) competitively in SH-SY5Y human neuroblastoma cells [15, 16], suggesting that *S. officinalis* L. may improve cognitive function and protect against memory deficits by increasing available acetylcholine levels. This study is therefore aimed at assessing the effect of the hydroethanolic leaf extract of *S. officinalis* L. on neuroinflammation and associated cognitive impairments.

## 2. Materials and Methods

### 2.1. Materials

Plexiglas chamber (Interplast Limited, Kumasi, Ghana) and elevated plus maze (EPM) box were used.

### 2.2. Chemicals

LPS from *Escherichia coli* (Servicebio Technology, Wuhan, China), ethanol (Fisher Scientific, Leicestershire, England), dimethyl sulfoxide (DMSO) (Fisher Scientific, Leicestershire, England), prednisolone (Taj Pharma, Mumbai, India), and ketamine (Hameln Pharma gmbh, Inselstraße 1, Germany) were used.

### 2.3. Experimental Animals

In this study, mice of the Institute of Cancer Research (ICR) species, both male and female, weighing 25–30 g and aged 8–10 weeks, were utilized. Animals were obtained from the Noguchi Memorial Institute of Medical Research in Legon, Accra. Mice were subsequently housed at the animal house facility of the Pharmacology Department at Kwame Nkrumah University of Science and Technology (KNUST). They were housed in groups of five in conventional 34 × 47 × 18 cm^3^ stainless-steel cages with bedding made of soft wood shavings in an Animal Biosafety Level 2 facility (ABSL2). The mice were given standard commercial pellet food and had unrestricted access to water, and the facility was lit for 12 h every day, from six in the morning to six in the evening. The animals were given 3 days to become used to their new environment before the experiment. Following this time of acclimation, the mouse random assignments were done to one of the experimental groups under investigation.

### 2.4. Plant Extraction

The leaves of *S. officinalis* L. were obtained from Exotic Penina Fields Group Ltd. in Nairobi, Kenya, a Global Good Agricultural Practices certified producer (GGN 4050373351410) and authenticated at the Department of Herbal Medicine, KNUST, where a voucher specimen was deposited (KNUST/HMI/2018/L011). *S. officinalis* L. was cultivated in well-drained volcanic soils at high-altitude with temperatures of 15°C–25°C, moderate humidity (50%–70%). It was harvested during the dry season (January–February) for optimal phytochemical content.

The leaves were meticulously cleaned, thoroughly rinsed in sterile distilled water, and completely dried using air. Subsequently, the plant material was finely grounded and stored in a clean, sealed container for later usage. With utmost precision, 40 g of the pulverized leaves was added to 200 mL of 70% ethanol [17] in a conical flask. Resulting mixture was left undisturbed for 24 h and then filtered using Whatman No. 1 filter paper. After allowing the filtrate to air dry at ambient temperature (~25°C) for 1 day, the extract was scraped off and weighed. The *S. officinalis* leaves had produced 6.0 g of extract, resulting in a percentage yield of 15% relative to the initial weight of the leaf material. Subsequently, the ethanolic *S. officinalis* L. leaf extract was suspended in 50% DMSO at a concentration of 10 mg/mL and stored in sterile bottles and referred to as SO. Ethanol (70%) was chosen to mimic the traditional preparation of the extract and its ability to extract both polar and moderately nonpolar phytochemicals, as supported by prior studies. Ethanol also minimizes potential toxicity in downstream biological studies.

### 2.5. Extract Characterization

A PerkinElmer Clarus 580 gas chromatography–Clarus SQ8S mass spectrometer system equipped with a capillary column of 30 m from the KNUST Central Laboratory was used to characterize the plant. The samples (1 *μ*L) were injected with a constant flow of helium at 2 mL/min and temperature of 230°C. Ethanol was used as the solvent. The oven temperature started at 80°C and was then gradually increased to 330°C at a rate of 15°C per minute. After reaching the desired temperature, it was held for 5 min. The mass spectrum was obtained using the full-scan method, which ranged from 33 to 600 m/z. The retention index was calculated using the n-alkane series, which served as the retention time standard [18].

### 2.6. General Phytochemical Screening

*S. officinalis* L. leaf extracts were screened for the presence of various phytochemicals. The crude extract was tested for the presence of alkaloids, glycosides, sterols, flavonoids, and saponins. Specific testing was conducted to confirm the existence of these bioactive substances following methods described by Aiyegoro and Okoh [19]. In order to determine whether tannins were present, a filtrate consisting plant extract (1.0 g) dissolved in 10 mL of distilled water was passed through a Whatman No. 1 filter paper along with a solution of ferric chloride reagent. Consequently, a blue tint was noticed, indicating the presence of tannins. On a steam bath, 0.5 g of the plant extract was dissolved in 5 mL of 1% hydrochloric acid (HCl) to look for alkaloids. One milliliter of the filtrate was then mixed with a few drops of Dragendorff's reagent. The mixture may include alkaloids if turbidity or precipitation starts to appear. In order to determine whether flavonoids were present, the extract (0.2 g) was heated after dissolving in 2 mL of methanol. Subsequently, a fragment of magnesium metal and a small quantity of concentrated HCl were added to the mixture. The manifestation of a red or orange hue serves as an indicator for the existence of flavonoids [19].

### 2.7. Toxicity Tests

#### 2.7.1. Acute Toxicity

Six groups of the animals with five mice each were given 0, 10, 100, 300, 1000, and 3000 mg/kg of the extracts, respectively [20]. Subsequently, the animals were continually observed, 1 h post administration and subsequently at 2-h interval, up to 24 h for lethality, weight changes, neurological functioning, respiration, changes in fur, normal functioning, and behavior [21].

#### 2.7.2. Irwin's Test

ICR mice received oral administration via gavage of the leaf extracts at doses of 0, 10, 100, 300, 1000, and 3000 mg/kg body weight. Each animal's behavioral, autonomic, and neurological conditions were assessed at 0, 15, 30, 60, 120, and 180 min and up to 24 h following administration [22, 23]. These included a range of physiological and behavioral changes, such as altered body posture, sedation/excitation, piloerection, ptosis, exophthalmos, abnormal behavior (e.g., forepaw treading, stereotypies, Straub tail), writhing, breathing rate, head twitches, jumping, tremor, and convulsions. The rating system consisted of a score of 0 = *normal activity* and 1, 2, 3, or 4 = *slight*, *moderate*, *extreme*, *or severe changes*, respectively (https://pspp.ninds.nih.gov/TestDescription/TestIrwin).

The animals were carefully handled beginning at the 15-min mark in order to note and record any changes in the following areas: grip strength; lacrimation; salivation; diarrhea; increased urination; increased defecation; vocalization; tail suspension; aggressiveness toward the handler; abdominal tone, limb tone, and reactivity to touch. The alterations in these parameters, including both magnitude and frequency, were appropriately recorded [22, 23].

### 2.8. LPS-Induced Neuroinflammation

#### 2.8.1. Grouping and Induction

Animals were divided into five (5) treatment groups (*n* = 8). Each animal was injected with LPS (500 *μ*g/kg of LPS in 0.5 mL), after which animals in the first group received 1 mL/kg of normal saline while the second group received oral prednisolone via gavage at 1 mg/kg daily for 3 days [24, 25]. The third, fourth, and fifth groups were also administered with 30, 100, and 300 mg/kg oral doses of *S. officinalis* L. leaf extracts, respectively, via gavage, once daily for 3 days [26]. The LPS was reconstituted in a vial disinfected with 70% alcohol and gauze, and the appropriate amount of solution was drawn up into the syringe. The animal was taken out of the cage and properly restrained in a head down position. To inject into the correct area of the abdomen, anatomical landmarks were identified. To protect the abdominal organs, for example, urinary bladder and cecum, the injection was administered in the lower right quadrant of the animal's abdomen. The needle was inserted with the bevel facing up into the injection site, horizontally at a 30°–40° angle, and to a depth where the entire bevel was within the abdominal cavity. Prior to injecting, the plunger was pulled back to ensure negative pressure, and when there was negative pressure, the injection proceeded. The needle was then pulled straight out and placed directly into a sharps container without being recapped. Animal was then returned to its cage and checked for any complications. Animals were injected with LPS once daily for 7 days to induce neuroinflammation.

#### 2.8.2. Object Recognition Test (ORT)

The recognition memory was assessed using the ORT. It evaluated the animal's ability to become familiar with a set of objects and then distinguish between a previously presented object and a new object in a subsequent test [27]. The animals were initially tested after receiving an IP injection of LPS but before receiving any medication. The ORT is divided into three phases: training, delay, and an object choice test.

The animals were exposed to a glass chamber (60 × 60 cm) for 10 min to acclimatize after which they were removed. The chamber was cleaned with 70% ethanol and after which a paper towel moistened with tap water was used to remove any lingering scent of mice which can confound results for the next batch of mice to be observed in the chamber. Oval-shaped containers were used in the experiment. The duplicate objects were green, while the novel object was blue. All the objects were 12.0 cm in size and could be easily climbed on to encourage exploration. The duplicate objects were put toward the corners of the two opposing ends of the chamber during the training phase (15 cm from each adjacent wall). After being brought into the glass chamber, the animal was given 5 min to explore various objects (A1 and A2). Afterwards, the animal was removed from the chamber for a period of 5 min or 2 h (delay period) and was then returned to the chamber for the novel object testing (5-min duration), where it was shown two objects in the same spatial locations as during training: one object (A3) was a third exact replica of the set of objects used during training, and the other object (B) was a novel object. On Day 2 of this study, the animals were trained and tested prior to the first dose of the intraperitoneal injection of LPS. On Day 6, the animals were trained and tested, 45 min after the injection of the last dose of the LPS but before the commencement of any treatment. Afterwards, the animals were trained and tested again on Day 9, after the treatment regimen.

Testing on Day 9 examined the animals following the completion of treatment. The animals were sacrificed on Day 10. For training and testing sessions, exploration periods were recorded and utilized to create a discriminating index: [time spent with object B − time spent with object A3]/[total time exploring both objects] [28]. Equal exploration of both objects is indicated by discrimination indices of 0 [27].

On the days of the test, the test was conducted in duplicate. The first one had a 5-min delay (time interval between the training and testing periods) while the second had a 2-h delay after the training. Positions of objects were counterbalanced between trials to eliminate location bias.

#### 2.8.3. EPM Test for Memory Evaluation

Two open arms (16 by 5 cm) and two covered arms (16 by 5 by 15 cm) made up the EPM. These arms were elevated from a central platform (5 by 5 cm). The whole maze was raised to a level 25 cm above the ground. On Day 1 of the experiment, animals were placed facing away from the center platform, at the end of an open arm. The length of time it took the animal to go from its open arm to one of its covered arms with all four legs was defined as the transfer latency (TL). The cut-off TL was set at 90 s. When an animal failed to enter one of the covered arms within 90 s, it was gently directed into a covered arm, and the TL stated as 90 s. Animals were then allowed to explore the maze for 2 more minutes before being returned to the cage. Twenty-four hours following the first trial, the memory or retention of this newly learnt task was assessed. This test was conducted on Day 2 before LPS administration, on Day 7, 45 min after LPS administration to induce neuroinflammation, and on Day 10, 1 h after the last dose of study treatments. The EPM test was conducted in a controlled testing room with low lighting (40–60 lux), quiet conditions (< 50 dB), and a stable temperature of 22°C–25°C with 40%–60% relative humidity. The maze was cleaned with 70% ethanol between trials to eliminate residual odors [29].

#### 2.8.4. Assay of TNF-*α* Expression in Brain Samples

To harvest the mouse brains, ketamine was administered at a dose of 80 mg/kg, i.p. to anesthetize the mice [30]. The anesthetized mice were placed in a supine position on a clean dissection surface with limbs secured. The surgical area was cleaned with 70% ethanol. A midline incision on the scalp to expose the skull was made. Using a dental drill, a small hole in the skull was created and enlarged toward the front. The brain was carefully lifted out of the cranial cavity, making sure to detach any remaining connections [31]. The extracted brain was placed in a suitable container and frozen at −80°C. The frozen whole hemispheres of the mouse brains were homogenized in cold PBS containing 100 mg tissue/mL and then centrifuged for 15 min at 12,000 *g*. The manufacturer's instructions were followed when using the ELISA kit to measure TNF-*α* levels in the brain. All TNF-*α* measurements were conducted in triplicates for accuracy, and TNF-*α* expression levels were normalized to the total protein content of brain homogenates. Outlier values were excluded based on Grubbs' test.

#### 2.8.5. Histopathological Analysis

Hematoxylin and eosin staining was performed on the mouse brain tissues to identify neuronal cell loss, glial cell proliferation, and mononuclear infiltration. The procedure was carried out as reported by Cardiff et al. [32]. The brain tissue was initially fixed, dehydrated, and subsequently embedded in paraffin wax. Subsequently, thin sections with a usual thickness of 4–6 *μ*m were obtained from the paraffin-embedded tissue using a microtome. The paraffin sections on the glass slides were placed in staining racks. Paraffin was removed from the samples in three rounds of xylene for 2 min each. The samples were hydrated by transferring them through a series of decreasing ethanol concentrations: 100%, 95%, and 70%. Afterwards, the slides were rinsed in running tap water for at least 2 min. To stain the samples, they were soaked in hematoxylin solution for 3 min, followed by thorough rinsing under running tap water. Then, they were stained in working eosin Y solution for 2 min. Dehydration was done by dipping the slides in 95% ethanol, transferring them through two rounds of 100% ethanol, and clearing them in three rounds of xylene. Finally, the slides were mounted with Permount and coverslips so they could be examined under a microscope [32].

### 2.9. Statistical Analysis

One-way ANOVA was used to examine the data, which was then subjected to Tukey's honest significant difference testing. GraphPad Prism Version 9.5.0 was used, and the results are provided as the mean ± SEM. *p* < 0.05 is required for statistical significance to be accepted.

## 3. Results

### 3.1. Extract Characterization

**Figure 1:**
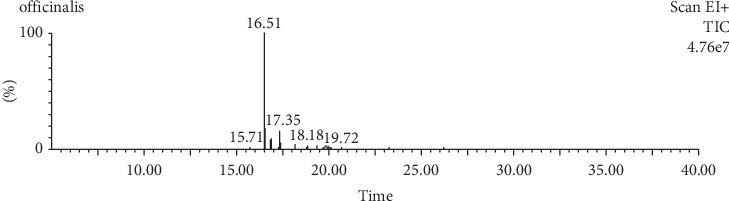
Chromatogram for *Salvia officinalis* L. leaves.

A hydroethanolic extract of *S. officinalis* L. was made and assessed for phytochemical composition using GC-MS to identify key phytoconstituents that may contribute to its pharmacological effects. The compound that made up the largest proportion, accounting for 74.87% of the total peak area, was identified as “8a(2H)-phenanthrenol, 7-ethenyldodecahydro-1,1,4a,7-tetramethyl-, acetate, [4as-(4a.alpha.,4b.beta.,7.beta.,8a.alpha.,10a.beta.)]”. Other significant compounds included “11,14,17-eicosatrienoic acid, methyl ester” (11.12%), “citronellyl butyrate” (6.92%), and “urea, 1-(4-fluorobenzoyl)-3-(4-methyl-6-trifluoromethylpyrimidin-2-yl)-” (3.16%). These findings indicate the presence of various organic compounds in *S. officinalis* L. leaves, such as terpenes, esters, and nitrogen-containing compounds. These compounds may contribute to the aroma, flavor, and bioactive properties of sage leaves. The results are shown in Table 1 and Figure 1.

### 3.2. Phytochemical Screening

The phytochemical screening of the hydroethanolic leaf extract of *S. officinalis* L. identified the presence of several important phytochemicals including alkaloids, glycosides, tannins, flavonoids, and coumarins. Saponins, triterpenoids, and phytosterols were found to be absent. Thus, the extract contains diverse phytochemicals like alkaloids, glycosides, and phenolic compounds which may be responsible for any pharmacological activities.

### 3.3. Toxicity Tests

The acute toxicity test results revealed that a single oral administration of *S. officinalis* L. leaf extract did not cause any significant observable adverse effects in the mice. Also, no mortalities were recorded at doses up to 3000 mg/kg of the leaf extract. Single dosing of the extracts in Irwin's test produced no changes in the behaviors of the animals that received 0, 10, 100, and 300 mg/kg throughout the experiment. For the 1000 mg/kg treatment group, all the animals had increased vocalization and were irritable to touch at 120 and 180 min. This dosing level also produced a Straub tail effect in the animals at 180 min. There was a delayed response to tail prick at the 180-min timestamp. In the 3000 mg/kg group, there was also increased vocalization and irritability to touch in all the animals from 120 to 180 min. In addition, Straub tail was observed in the animals at 120 and 180 min. Also, delayed response to tail prick was observed in the animals at the 180-min time point. No mortality was recorded in any of the treatment groups for up to 24 h.

### 3.4. Effect of *S. officinalis* on LPS-Induced Neuroinflammation

#### 3.4.1. Recognition Memory in Mice Before and After LPS Administration

This section presents data on the evaluation of the effect of *S. officinalis* extract on recognition memory using the ORT that measures preference for novel objects. In the ORT, the discrimination index (DI) reflects the preference for novel objects over familiar ones. A transition to negative DI values in LPS-treated mice indicates impaired recognition memory, as the mice spend more time exploring previously encountered objects rather than showing a preference for novel ones.

**Figure 2:**
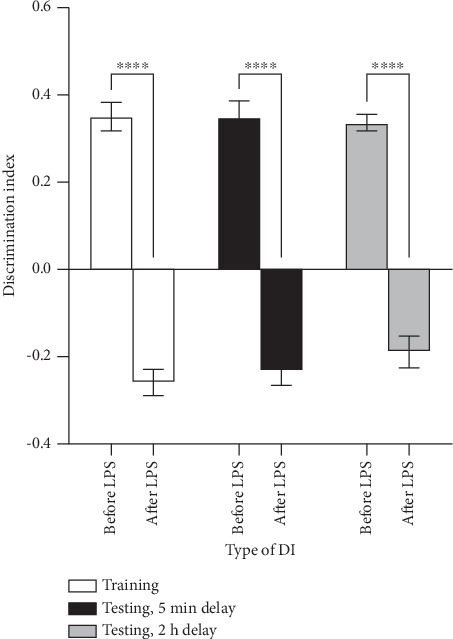
Discrimination index before and after LPS administration in the ORT. The values are presented as means ± SEM. ⁣^∗∗∗∗^*p* < 0.0001 when comparisons were made before and after LPS administration by multiple comparison using mixed-effects analysis. DI = discrimination index; ORT = object recognition test.

Before the administration of LPS, positive DI values were recorded across the time intervals with no significant differences among them. Following the administration of LPS, the DI values were predominantly negative across all groups, with no statistically significant differences among them. There was however a significant change (*p* < 0.0001) in DI when observations before and after LPS administration were compared in the ORT (Figure 2).

### 3.5. Effect of *S. officinalis* L. on Recognition Memory in Mice

**Figure 3:**
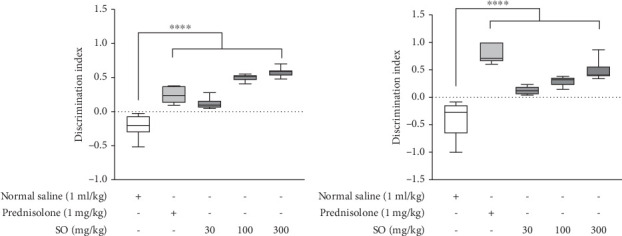
Effect of *S. officinalis* on discrimination index in mice after (a) 5-min and (b) 2-h delay. The values are presented as mean ± SEM. ⁣^∗∗∗∗^*p* < 0.0001, when treatments were compared with normal saline–treated control (one-way ANOVA followed by Dunnett's post hoc test). DI = discrimination index.

After treatments, the normal saline control mice maintained negative DIs. The *S. officinalis* L.–treated mice (30, 100, 300 mg/kg) showed improvement in DI compared to control, shifting toward positive values. In the 5-min delay period, all the other groups apart from the normal saline control mice recorded positive DI values. There were statistically significant differences in DI between the normal saline control group and *S. officinalis* L.–treated mice at all studied doses (30, 100, 300 mg/kg) (*p* < 0.0001). Significant difference in DI was also observed when normal saline control mice were compared with prednisolone-treated rats (*p* < 0.0001) as seen in Figure 3a.

After the 2-h delay, there was 22.5% decrease in DI of normal saline control mice from the baseline while that of *S. officinalis* L. groups (30, 100, 300 mg/kg) increased by 31.17%, 46.75%, and 65.5%, respectively. There was positive and significant DI recorded for *S. officinalis* L. at 30 mg/kg (*p* < 0.0001), 100 mg/kg (*p* < 0.0001), and 300 mg/kg (*p* < 0.0001). Prednisolone recorded the highest change in DI from the baseline training value (96.50%) (Figure 3b).

Following the 5-min delay after the study treatments, the normal saline–treated control mice had mean DI of −0.213 ± 0.057. In contrast, the *S. officinalis* L.–treated mice recorded positive mean DI, ranging from 0.11 ± 0.04 to 0.57 ± 0.02. These increases in DI were statistically significant (*p* < 0.001) when compared to the normal saline–treated control mice. The prednisolone group had mean DI of 0.24 ± 0.05, which was also statistically significant (*p* < 0.001).

During the 2-h delay post treatment period, the normal saline–treated control mice had the lowest mean DI value of −0.40 ± 0.11. The treatment groups receiving *S. officinalis* L. showed positive mean DI ranging from 0.13 ± 0.030 to 0.48 ± 0.06. These increases in DI were statistically significant (*p* < 0.001) when compared to the normal saline–treated control. Similarly, the prednisolone group had mean DI value of 0.78 ± 0.07, and the *p* value was highly statistically significant (*p* < 0.001). Taken together, treatment with *S. officinalis* L. resulted in improvements in discrimination ability compared to the control as shown in Table 2.

### 3.6. Spatial Memory in Mice Before and After LPS Administration

**Figure 4:**
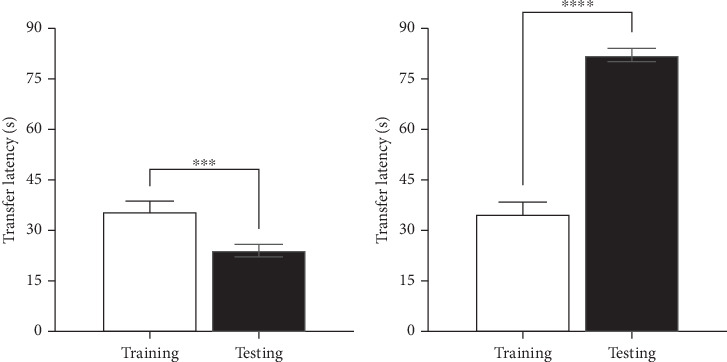
Transfer latency (a) before and (b) after LPS administration in the EPM test. The values are presented as mean ± SEM. ⁣^∗∗∗^*p* ≤ 0.0001, when training compared with testing (Student's *t*-test). EPM = elevated plus maze.

To assess spatial memory performance, the EPM test was used to determine changes in risk attitude and exploratory behavior, both of which are influenced by memory and anxiety-related processes. A decrease in TL (the time taken to enter the closed arm) indicates improved spatial memory, as the animal recalls the previously learned safe environment, whereas an increase in TL suggests impaired memory retention or heightened anxiety. In the EPM before LPS administration, transfer latencies generally decreased from training to testing in all the mice. The decrease in TL from training to testing is statistically significant (*p* = 0.0001) (Figure 4a). After LPS administration, the transfer latencies were markedly higher in the testing phase compared to initial training in all the mice studied (Figure 4b).

There was 12.6% reduction in testing transfer latencies compared to that of training before LPS. Training transfer latencies however increased by 52.3% compared to testing transfer latencies post LPS. These percentage changes in TL before and after LPS administration were statistically significant (*F*(1, 38) = 162.8, *p* < 0.0001).

### 3.7. Effect of *S. officinalis* L. on Spatial Memory in Mice

After treatment, the control group showed a decrease in mean TL from 71.16 ± 6.57 during training to 64.37 ± 10.54 s during testing. In contrast, the treatment groups receiving different doses of *S. officinalis* L. demonstrated further reductions in mean TL. The mean TL values for *S. officinalis* L. 30–300 mg/kg groups were 56.39 ± 15.45, 27.68 ± 6.16, and 26.09 ± 03.03 s, respectively, during the testing phase. Notably, the *p* values associated with the *S. officinalis* L. 100 and 300 mg/kg groups were statistically significant (*F*(4, 35) = 10.57, *p* < 0.05), indicating a significant difference compared to the control. On the other hand, the prednisolone group exhibited the lowest mean TL of 18.05 ± 3.90 s, and the *p* value was highly statistically significant (*F*(4, 35) = 4.29, *p* = 0.0079). In summary, after treatment, the *S. officinalis* L.–treated groups demonstrated significant reductions in TL compared to the control, with 100 and 300 mg/kg exhibiting the most pronounced effect. Nonetheless, prednisolone showed the most significant reduction in TL (Table 3).

### 3.8. Effect of *S. officinalis* L. on TNF-*α* Expression in Mouse Brain Tissues

**Figure 5:**
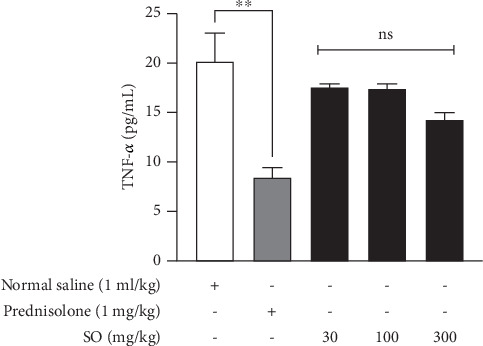
Effect *S. officinalis* treatment on brain tissue expression of TNF-*α*. The values are presented as mean ± SEM. ^NS^*p* > 0.05, ⁣^∗∗^*p* = 0.0019, when compared with normal saline–treated control (one-way ANOVA followed by Dunnett's post hoc test). TNF-*α* = tumor necrosis factor-alpha.

Normal saline–treated control mice exhibited TNF-*α* level of 20.17 ± 2.85 pg/mL. After SO treatment at 30, 100, and 300 mg/kg, mice showed brain tissue TNF-*α* levels of 17.51 ± 0.57, 17.37 ± 0.93, and 14.20 ± 1.30 pg/mL, respectively. However, at the doses of SO studied, the observed reduction in tissue levels of TNF-*α* was not statistically significant compared with the normal saline–treated control mice. Prednisolone administration resulted in the mean TNF-*α* level reducing to 8.44 ± 1.71 pg/mL (*F* (3, 36) = 5.35, *p* = 0.004) (Figure 5).

### 3.9. Effect of *S. officinalis* on Histopathological Parameters of Brain Tissues

**Figure 6:**
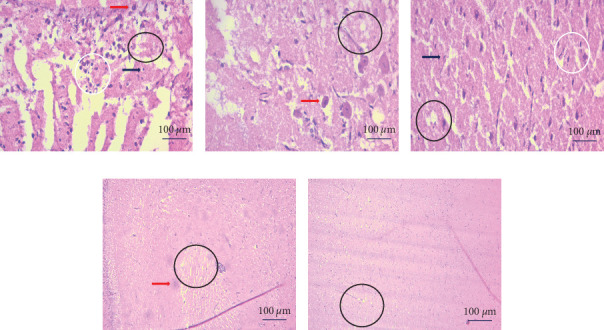
Effect of *S. officinalis* on brain histopathology of mice with neuroinflammation. (a) Normal saline–treated control. (b) Prednisolone-treated mice. (c) 30 mg/kg SO–treated mice. (d) 100 mg/kg SO–treated mice. (e) 300 mg/kg SO–treated mice. Red arrow: apoptotic neurons; blue arrow: periventricular mononuclear infiltration; black circle: oedema; white circle: gliosis. The micron bar represents 100 *μ*m.

The normal saline–treated control mice (Figure 6a) showed extensive neuronal cell loss (red arrow), along with gliosis (proliferation of glial cells) (white circle) and a focal mononuclear infiltrate (presence of plasma cells and lymphocytes) (blue arrow) in the cerebellum, pons, hippocampus, cortex, and lateral ventricles. Mice treated with prednisolone (Figure 6b) exhibited few apoptotic neurons, a scanty periventricular mononuclear infiltrate, and no gliosis or neuronal cell loss in the cerebellum, pons, hippocampus, and cortex. Mice treated with 30 mg/kg SO (Figure 6c) showed focal neuronal cell loss, a few apoptotic neurons, and oedema in the cerebellum (black circle), cortex, and hippocampus. At 100 mg/kg of SO (Figure 6d), mice showed apoptotic neuronal cells, a mild perivascular mononuclear infiltrate, and focal mild neuronal cell loss in the cortex, hippocampus, cerebellum, and pons. Mice receiving 300 mg/kg of SO (Figure 6e) did not show any significant histological lesions, with no gliosis, mononuclear infiltrates, apoptotic or necrotic neurons in the cerebellum and pons, but there was a small focus of oedema with occasional mononuclear cells noted in the cortex (Figure 6).

## 4. Discussion

In this study, the acute toxicity evaluation of *S. officinalis* L. leaf extracts in the mice revealed no adverse effects or mortality up to a high dose of 3000 mg/kg. This suggests that *S. officinalis* L. leaf extracts have a high safety margin and a low toxicity risk when administered orally [33, 34]. The Irwin test, which assesses potential neurological and behavioral effects more thoroughly, revealed that, at lower doses, no changes were observed in the animals. This indicates that the neurological and behavioral functions of the animals were not noticeably affected by these doses. At 1000 mg/kg, some animals showed increased vocalization, irritability, and the Straub tail effect, indicating mild neurological excitation. These findings indicate that animals may experience mild neurological excitement when given a higher dosage of *S. officinalis* L. leaf extract. At 3000 mg/kg, similar effects were observed, as well as a slightly delayed pain response, indicating moderate neurological excitation [22]. This suggests that the extracts of *S. officinalis* L. leaves may have a stronger effect on neurological function at this specific dose. The effects, however, were mild to moderate and transient, with no fatalities. This confirms the general safety of acute *S. officinalis* L. extract administration.

The optimal therapeutic doses identified were 100–300 mg/kg, which showed no adverse effects and are significantly lower than the doses causing observable neuroexcitation. More chronic toxicity research can shed light on the long-term safety of repeated dosing. Overall, these toxicity studies show that *S. officinalis* L. leaf extracts are relatively nontoxic in the short term, with potential adverse neurological effects appearing only at very high supratherapeutic doses. This emphasizes their advantage in terms of safety over conventional synthetic drugs.

The ORT was used to assess recognition memory in this study. Recognition memory is a type of declarative memory that is dependent on the integrity of structures in the medial temporal lobe, including the hippocampus [35]. Systemic LPS administration causes neuroinflammation, which results in the release of proinflammatory cytokines such as IL-1*β*, IL-6, and TNF-*α*, which disrupt hippocampal synaptic plasticity through effects on NMDA receptor signaling. Long-term potentiation, neurogenesis, and spatial memory formation are all impaired as a result [36]. *S. officinalis* L. treatment resulted in improvements in DI, indicating improvements in recognition memory disruptions possibly caused by neuroinflammatory cytokine signaling. This could be accomplished by reducing microglial activation and subsequent cytokine release. Notably, the highest *S. officinalis* L. dose demonstrated resolution of recognition memory impairment comparable to that of prednisolone, a corticosteroid drug that effectively suppresses neuroinflammation by transrepressing NF-*κ*B target genes encoding cytokines, adhesion molecules, and other mediators [37]. The similar observable effects suggest that at sufficient doses, *S. officinalis* L. can mimic the antineuroinflammatory effects of corticosteroid drugs.

The EPM was employed to assess spatial long-term memory, which is dependent on hippocampal function [38]. TL is a metric that measures the time it takes to move from the open arm to the closed arm, with lower latency indicating better spatial memory. LPS is known to cause neuroinflammation and impairs spatial memory consolidation and retrieval by disrupting hippocampal synaptic plasticity [36]. *S. officinalis* L. treatment after LPS significantly reduced TL with significant reductions at the higher doses. This suggests that taking *S. officinalis* L. after LPS exposure can reduce neuroinflammation-induced hippocampal synaptic dysfunction and effectively restore spatial memory [39]. Ultimately, *S. officinalis* L. has efficacy in restoring spatial memory that has been compromised by neuroinflammation, most likely by targeting neuroinflammatory signaling cascades that disrupt hippocampal synaptic plasticity. This adds to the evidence that *S. officinalis* L. has the potential to be a natural alternative for treating cognitive deficits associated with neuroinflammatory conditions.

TNF-*α* is a proinflammatory cytokine that mediates neuroinflammation and contributes to cognitive impairment in conditions such as AD [40]. The administration of LPS causes neuroinflammation characterized by elevated TNF-*α* levels [36]. Carnosol, carnosic acid, and rosmarinic acid are compounds found in *S. officinalis* L. that can inhibit TNF-*α* production by downregulating NF-*κ*B signaling [41]. *S. officinalis* L. however exhibited limited effect against TNF-*α* levels. This may be due to the doses of *S. officinalis* L. used in the experiment. Prednisolone was more effective than *S. officinalis* L. in lowering TNF-*α* levels.

The histological report from this study revealed widespread neuronal damage in the control and the lower *S. officinalis* L. dose groups, accompanied by gliosis. This suggests that the LPS-induced neuroinflammation had detrimental effects on the neurons in the brain. Gliosis refers to the activation of glial cells, particularly microglia, in response to neuroinflammation [42]. The mononuclear infiltration consisting of plasma cells and lymphocytes observed is also suggestive of an immune response to neuronal damage. This immune response is characterized by the recruitment and activation of immune cells to the site of injury in order to initiate the repair process and eliminate any potential threats [43]. The prednisolone and highest *S. officinalis* L. dose groups showed minimal pathological changes suggestive of a protective effect on the neurons, preventing damage or promoting repair mechanisms. Prednisolone as a corticosteroid is known to effectively reduce inflammation and suppresses immune response [44]. These findings indicate that *S. officinalis* L. has an effect on neuronal health in mice. While low and moderate doses of *S. officinalis* L. were associated with varying degrees of neuronal damage, the highest dose appeared to protect against these pathological changes.

The chemical profiling of *S. officinalis* leaf extracts using GC-MS analysis also revealed valuable information about the plant. The most abundant component found was 8a(2H)-phenanthrenol, 7-ethenyldodecahydro-1,1,4a,7-tetramethyl-acetate (74.87%), also known as carnosic acid. This phenolic diterpene has anti-inflammatory, antioxidant, and neuroprotective properties [45]. It inhibits proinflammatory cytokines such as TNF-*α* and activates antioxidant signaling via Nrf2 [46]. This most likely contributes significantly to *S. officinalis* L.'s anti-neuroinflammatory efficacy observed in this study. Another significant component was 11,14,17-eicosatrienoic acid, methyl ester (11.12%), a fatty acid derivative. Polyunsaturated fatty acids, such as eicosapentaenoic acid, have anti-inflammatory properties in the brain and can reduce neuroinflammation-related cognitive impairment [47]. Citronellyl butyrate (6.92%), a monoterpenoid ester that inhibits NO and PGE2 production [48], was also identified as an anti-inflammatory compound [49]. The presence of such a diverse array of bioactive anti-inflammatory phytochemicals likely explains *S. officinalis* L.'s observed efficacy in suppressing neuroinflammatory signaling and restoring cognitive function. Multitarget mechanisms provide advantages over single-target drugs. Individual component isolation and testing can provide more information about their specific neuropharmacological properties.

In addition to these, the phytochemical screening of *S. officinalis* L. extract revealed that it contains several bioactive compounds that are likely to contribute to the anti-neuroinflammatory effects observed in this study. Tannins were found in the extract. Tannins such as gallic acid and pentagalloylglucose have anti-inflammatory effects due to their ability to inhibit NF-*κ*B-mediated cytokine production [50, 51]. Glycosides were also found. Salvianolic acids are glycosides found in *S. officinalis* L. that exhibit neuroprotective properties through antioxidant, anti-inflammatory, and antiapoptotic mechanisms [52]. The extract contained alkaloids that were discovered. *S. officinalis* L. contains alkaloids, which are important bioactive compounds [9]. These alkaloids are the largest group of plant secondary metabolites and have various pharmacological activities, such as anti-inflammatory properties [53]. Flavonoids were also detected. Luteolin and quercetin are flavonoids found in *S. officinalis* L. [54]. They can repress microglial activation and its corresponding proinflammatory cytokine release [55]. Coumarins were found. Scopoletin is a coumarin that can cross the blood–brain barrier and inhibit iNOS, COX-2, and TNF-*α* to reduce neuroinflammation [56]. The synergistic multitarget action of these diverse anti-inflammatory phytochemicals mediates the efficacy of *S. officinalis* L. extract in mitigating neuroinflammation and associated cognitive impairment. Individual phytochemical isolation and testing can provide more information about their neuropharmacology.

The cognitive and behavioral improvements observed following *S. officinalis* extract treatment have been attributed to its anti-inflammatory effects. However, we acknowledge that neurotransmitter modulation and anti-inflammatory activity could both contribute to produce these effects. Multiple studies have shown that *S. officinalis* along with its bioactive constituents like phenolic diterpenes and flavonoids block the action of major brain enzymes AChE, monoamine oxidase (MAO), and catechol-O-methyltransferase (COMT) [57–59]. Increased availability of acetylcholine due to AChE inhibition plays a fundamental role in learning and memory [60] potentially leading to better discrimination indices in the ORT. The exploratory activity along with risk-taking behavior in the object recognition and EPM tests could be altered by COMT and MAO-modulated dopamine signaling [61, 62]. In this study, cognitive improvements were observed even at lower extract doses, where reductions in TNF-*α* levels and histopathological changes were less pronounced. This suggests that *S. officinalis* may enhance memory and behavior through both anti-inflammatory and neurotransmitter-related pathways. Future studies incorporating additional assays, such as ex vivo neurotransmitter quantification or enzyme inhibition assays, will be necessary to further describe these mechanisms.

Another limitation of our study is the absence of a control group receiving *S. officinalis* extract alone, without LPS treatment. The analysis of data from an additional control group receiving *S. officinalis* extract alone would have allowed researchers to distinguish between neuroinflammatory protection and independent procognitive properties of the extract. Future research addressing this question must incorporate *S. officinalis* extract treatment without LPS exposure to prove how the plant produces protective or cognitive improvement outcomes. Further, another limitation arises from the lack of TNF-*α* measurements in untreated control mice receiving LPS treatment. Studies show that LPS treatment causes TNF-*α* elevation in brain tissue [36, 40, 42]. But our experimental design could have confirmed LPS neuroinflammation better by directly measuring baseline TNF-*α* amounts. Future research should include a control group receiving no LPS treatment to identify base TNF-*α* levels to better understand the mechanism of action of *S. officinalis*.

## 5. Conclusion

The present work demonstrated that neuroinflammation modulating *S. officinalis* L. leaf extract improves memory. When administered after the LPS treatment, *S. officinalis* L. enhanced the memory and cognitive functions of mice. GC-MS and phytochemical analysis revealed the presence of bioactive compound that may explain the observed effects. From the results of the acute toxicity studies performed, *S. officinalis* L. possesses a relatively desirable safety consideration. *S. officinalis* L. therefore has potential pharmacological activity of neuroinflammatory diseases and requires further research as a potential natural therapeutic agent.

## Figures and Tables

**Table 1:** GC-MS results of *Salvia officinalis* L. leaf extract.

**RT**	**Area**	**% area**	**Name**
15.711	26,980.0	1.53	Methyl-6-deoxy-6-fluoro-2,3,4-tri-O-methylád-galactopyranoside
16.511	1,323,420.8	74.87	8a(2H)-Phenanthrenol, 7-ethenyldodecahydro-1,1,4a,7-tetramethyl-, acetate, [4as-(4a.alpha.,4b.beta.,7.beta.,8a.alpha.,10a.beta.)]-
16.871	122,276.8	6.92	Citronellyl butyrate
17.281	7652.2	0.43	Cyclopropane, (2-methylenebutyl)-
17.351	196,636.3	11.12	11,14,17-Eicosatrienoic acid, methyl ester
18.181	55,791.4	3.16	Urea, 1-(4-fluorobenzoyl)-3-(4-methyl-6-trifluoromethylpyrimidin-2-yl)-
19.001	16,694.4	0.94	Phthalic acid, neopentyl 4-nitrophenyl ester
20.251	10,682.2	0.60	Hexanoic acid, 2-ethyl-, 2-octyldodecyl ester
36.025	7468.3	0.42	Formaldehyde, dimethylhydrazone

*Note:* Area (total peak area) = abundance; % area = relative abundance.

Abbreviation: RT, retention time.

**Table 2:** Statistical significance of mean DI between treatment groups.

**Study phase**	**Treatment group**	**DI** **M** **e** **a** **n** ± **SEM**	**p** ** value**
After treatment			
Training			
Control		−0.174 ± 0.037	
	Prednisolone	0.287 ± 0.067	< 0.0001
	SO 30 mg/kg	0.192 ± 0.056	0.0003
	SO 100 mg/kg	0.283 ± 0.056	< 0.0001
	SO 300 mg/kg	0.328 ± 0.043	< 0.0001

Testing 5-min delay			
Control		−0.213 ± 0.057	
	Prednisolone	0.240 ± 0.049	< 0.0001
	SO 30 mg/kg	0.113 ± 0.036	< 0.0001
	SO 100 mg/kg	0.500 ± 0.017	< 0.0001
	SO 300 mg/kg	0.565 ± 0.024	< 0.0001

Testing 2-h delay			
Control		−0.399 ± 0.113	
	Prednisolone	0.778 ± 0.071	< 0.0001
	SO 30 mg/kg	0.125 ± 0.030	0.0002
	SO 100 mg/kg	0.294 ± 0.030	< 0.0001
	SO 300 mg/kg	0.481 ± 0.061	< 0.0001

Abbreviation: DI, discrimination index.

**Table 3:** Mean transfer latency after treatment in the EPM test.

**Study phase**	**Treatment group**	**Transfer latency** **M** **e** **a** **n** ± **SEM**	**p** ** value**
After treatment			
Training			
Control		71.160 ± 6.568	
	Prednisolone	29.790 ± 8.441	0.0229
	SO 30 mg/kg	54.570 ± 13.210	0.6928
	SO 100 mg/kg	30.780 ± 7.401	0.0198
	SO 300 mg/kg	33.390 ± 9.856	0.0329

Testing			
Control		64.370 ± 10.540	
	Prednisolone	18.05 ± 3.897	0.0079
	SO 30 mg/kg	56.390 ± 15.450	0.9684
	SO 100 mg/kg	27.680 ± 6.162	0.0373
	SO 300 mg/kg	26.09 ± 03.031	0.0274

Abbreviation: EPM, elevated plus maze.

## Data Availability

The data that support the findings of this study are available from the corresponding authors upon reasonable request.

## References

[1] Heneka M. T., Carson M. J., El Khoury J. (2015). Neuroinflammation in Alzheimer’s disease. *The Lancet Neurology*.

[2] Hirsch E. C., Hunot S. (2009). Neuroinflammation in Parkinson’s disease: a target for neuroprotection?. *The Lancet Neurology*.

[3] Valcour V. G. (2016). HIV, aging, and cognition: emerging issues. *Topics in Antiviral Medicine*.

[4] DiSabato D., Quan N., Godbout J. P. (2016). Neuroinflammation: the devil is in the details. *Journal of Neurochemistry*.

[5] Skrzypczak-Wiercioch A., Sałat K. (2022). Lipopolysaccharide-induced model of neuroinflammation: mechanisms of action, research application and future directions for its use. *Molecules*.

[6] Ciesielska A., Matyjek M., Kwiatkowska K. (2021). TLR4 and CD14 trafficking and its influence on LPS-induced pro-inflammatory signaling. *Cellular and Molecular Life Sciences*.

[7] Liu T., Zhang L., Joo D., Sun S. C. (2017). NF-*κ*B signaling in inflammation. *Signal Transduction and Targeted Therapy*.

[8] Ben-Akacha B., Ben Hsouna A., Generalić Mekinić I. (2023). *Salvia officinalis* L. and *Salvia sclarea* essential oils: chemical composition, biological activities and preservative effects against Listeria monocytogenes inoculated into minced beef meat. *Plants*.

[9] Ghorbani A., Esmaeilizadeh M. (2017). Pharmacological properties of Salvia officinalis and its components. *Journal of Traditional and Complementary Medicine*.

[10] Kamatou G. P. P., Makunga N. P., Ramogola W. P. N., Viljoen A. M. (2008). South African Salvia species: a review of biological activities and phytochemistry. *Journal of Ethnopharmacology*.

[11] Naomi R. B., Mwanarusi S., Musyoka I. F. (2014). Effects of nitrogen, phosphorus and irrigation regimes on growth and leaf productivity of sage (Salvia officinalis L.) in Kenya. *Annals of Biological Research*.

[12] Arranz E., Jaime L., Lopez M. C., Vicente G., Reglero G., Santoyo S. (2014). Supercritical sage extracts as anti-inflammatory food ingredients. *Industrial Crops and Products*.

[13] Kolac U. K., Ustuner M. C., Tekin N., Ustuner D., Colak E., Entok E. (2017). The anti-inflammatory and antioxidant effects of *Salvia officinalis* on lipopolysaccharide-induced inflammation in rats. *Journal of Medicinal Food*.

[14] Eidi M., Eidi A., Bahar M. (2006). Effects of *Salvia officinalis* L. (sage) leaves on memory retention and its interaction with the cholinergic system in rats. *Nutrition*.

[15] Altınterim B. (2015). Cholinergic effects of sage (*Salvia officinalis* l.) leaves. *Erzincan University Journal of Science and Technology*.

[16] Savelev S., Okello E., Perry N. S. L., Wilkins R. M., Perry E. K. (2003). Synergistic and antagonistic interactions of anticholinesterase terpenoids in Salvia lavandulaefolia essential oil. *Pharmacology Biochemistry and Behavior*.

[17] Rudhani I. (2018). In vitro antibacterial properties of ethanol extract from *Salvia officinalis* (L.) plant growing wild in Kosovo. *Biomedical Journal of Scientific & Technical Research*.

[18] Vieira N. M., Vital C. E., Pontes C. S. L. (2019). Method for the metabolic profile of plant tissues by gas chromatography coupled to mass spectrometry (GC/MS). Protocols. https://www.protocols.io/view/method-for-the-metabolic-profile-of-plant-tissues-8suhwew.

[19] Aiyegoro O. A., Okoh A. I. (2010). Preliminary phytochemical screening and in vitro antioxidant activities of the aqueous extract of Helichrysum longifolium DC. *BMC Complementary and Alternative Medicine*.

[20] European Medicines Agency (2016). Assessment report on *Salvia officinalis* L., folium and *Salvia officinalis* L., aetheroleum. https://www.ema.europa.eu/en/documents/herbal-report/draft-assessment-report-salvia-officinalis-l-folium-salvia-officinalis-l-aetheroleum_en.pdf.

[21] Di L., Kerns E. H. (2016). *Drug-Like properties: Concepts, structure design and methods: From ADME to toxicity optimization*.

[22] Irwin S. (1968). Comprehensive observational assessment: Ia. A systematic, quantitative procedure for assessing the behavioral and physiologic state of the mouse. *Psychopharmacologia*.

[23] Williams M., Porsolt R. D., Lacroix P., Enna S. J., David B. B. (2007). Safety pharmacology II-CV, GI, respiratory and renal safety. *xPharm: The Comprehensive Pharmacology Reference*.

[24] Bruscoli S., Febo M., Riccardi C., Migliorati G. (2021). Glucocorticoid therapy in inflammatory bowel disease: mechanisms and clinical practice. *Frontiers in Immunology*.

[25] Quattrocelli M., Wintzinger M., Miz K. (2022). Intermittent prednisone treatment in mice promotes exercise tolerance in obesity through adiponectin. *Journal of Experimental Medicine*.

[26] Miroddi M., Navarra M., Quattropani M. C., Calapai F., Gangemi S., Calapai G. (2014). Systematic review of clinical trials assessing pharmacological properties of salvia species on memory, cognitive impairment and Alzheimer’s disease. *CNS Neuroscience & Therapeutics*.

[27] Tyor W. R., Bimonte-Nelson H. (2018). A mouse model of HIV-associated neurocognitive disorders: a brain-behavior approach to discover disease mechanisms and novel treatments. *Journal of Neurovirology*.

[28] Lueptow L. M. (2017). Novel object recognition test for the investigation of learning and memory in mice. *Journal of Visualized Experiments: JoVE*.

[29] Dhingra D., Kumar V. (2012). Memory-enhancing activity of palmatine in mice using elevated plus maze and Morris water maze. *Advances in Pharmacological Sciences*.

[30] University of Michigan Animal Care and Use (2023). Guidelines on Anesthesia and Analgesia in Mice. https://az.research.umich.edu/animalcare/guidelines/guidelines-anesthesia-and-analgesia-mice.

[31] Spijker S., Li K. (2011). Dissection of rodent brain regions. *Springer nature experiments*.

[32] Cardiff R. D., Miller C. H., Munn R. J. (2014). Manual hematoxylin and eosin staining of mouse tissue sections. *Cold Spring Harbor Protocols*.

[33] Chan K. (2003). Some aspects of toxic contaminants in herbal medicines. *Chemosphere*.

[34] Li X., Luo Y., Wang L. (2010). Acute and subacute toxicity of ethanol extracts from *Salvia przewalskii* Maxim in rodents. *Journal of Ethnopharmacology*.

[35] Antunes M., Biala G. (2012). The novel object recognition memory: neurobiology, test procedure, and its modifications. *Cognitive Processing*.

[36] Akiyama H., Barger S., Barnum S. (2000). Inflammation and Alzheimer’s disease. *Neurobiology of Aging*.

[37] Coutinho A. E., Chapman K. E. (2011). The anti-inflammatory and immunosuppressive effects of glucocorticoids, recent developments and mechanistic insights. *Molecular and Cellular Endocrinology*.

[38] Itoh J., Nabeshima T., Kameyama T. (1990). Utility of an elevated plus-maze for the evaluation of memory in mice: effects of nootropics, scopolamine and electroconvulsive shock. *Psychopharmacology*.

[39] Miroddi M., Calapai G., Calapai F. (2014). Potential beneficial effects of sage (*Salvia officinalis* L.) in the prevention and treatment of Alzheimer’s disease. *Journal of Ethnopharmacology*.

[40] Decourt B., Lahiri D. K., Sabbagh M. N. (2017). Targeting tumor necrosis factor alpha for Alzheimer’s disease. *Current Alzheimer Research*.

[41] Farhat M. B., Landoulsi A., Chaouch-Hamada R., Sotomayor J. A., Jordán M. J. (2013). Characterization and quantification of phenolic compounds and antioxidant properties of Salvia species growing in different habitats. *Industrial Crops and Products*.

[42] Zhao J., Bi W., Xiao S. (2019). Neuroinflammation induced by lipopolysaccharide causes cognitive impairment in mice. *Scientific Reports*.

[43] Alam A., Thelin E. P., Tajsic T. (2020). Cellular infiltration in traumatic brain injury. *Journal of Neuroinflammation*.

[44] Stahn C., Buttgereit F. (2008). Genomic and nongenomic effects of glucocorticoids. *Nature Clinical Practice Rheumatology*.

[45] Berdowska I., Zieliński B., Fecka I., Kulbacka J., Saczko J., Gamian A. (2013). Cytotoxic impact of phenolics from *Lamiaceae* species on human breast cancer cells. *Food Chemistry*.

[46] Mirza F. J., Zahid S., Holsinger R. M. D. (2023). Neuroprotective effects of carnosic acid: insight into its mechanisms of action. *Molecules*.

[47] Xia J., Yang L., Huang C. (2023). Omega-3 polyunsaturated fatty acid eicosapentaenoic acid or docosahexaenoic acid improved ageing-associated cognitive decline by regulating glial polarization. *Marine Drugs*.

[48] de Santana M. T., de Oliveira M. G. B., Santana M. F. (2013). Citronellal, a monoterpene present in Java citronella oil, attenuates mechanical nociception response in mice. *Pharmaceutical Biology*.

[49] Armanini D., Sabbadin C., Donà G., Clari G., Bordin L. (2014). Aldosterone receptor blockers spironolactone and canrenone: two multivalent drugs. *Expert Opinion on Pharmacotherapy*.

[50] Piazza S., Fumagalli M., Martinelli G. (2022). Hydrolyzable tannins in the management of Th1, Th2 and Th17 inflammatory-related diseases. *Molecules*.

[51] Tong J., Fang J., Zhu T. (2021). Pentagalloylglucose reduces AGE-induced inflammation by activating Nrf2/HO-1 and inhibiting the JAK2/STAT3 pathway in mesangial cells. *Journal of Pharmacological Sciences*.

[52] López V., Martín S., Gómez-Serranillos M. P., Carretero M. E., Jäger A. K., Calvo M. I. (2010). Neuroprotective and neurochemical properties of mint extracts. *Phytotherapy Research: PTR*.

[53] Souto A. L., Tavares J. F., da Silva M. S., Diniz M. F. F. M., de Athayde-Filho P. F., Filho J. M. B. (2011). Anti-inflammatory activity of alkaloids: an update from 2000 to 2010. *Molecules*.

[54] Iacopetta D., Ceramella J., Scumaci D. (2023). An update on recent studies focusing on the antioxidant properties of Salvia species. *Antioxidants*.

[55] Spencer J. P. E. (2009). The impact of flavonoids on memory: physiological and molecular considerations. *Chemical Society Reviews*.

[56] Zhang F., Zhang Y., Yang T. (2019). Scopoletin suppresses activation of dendritic cells and pathogenesis of experimental autoimmune encephalomyelitis by inhibiting NF-*κ*B signaling. *Frontiers in Pharmacology*.

[57] Lopresti A. L. (2017). Salvia (sage): a review of its potential cognitive-enhancing and protective effects. *Drugs in R&D*.

[58] Margetts G., Kleidonas S., Zaibi N. S., Zaibi M. S., Edwards K. D. (2022). Evidence for anti-inflammatory effects and modulation of neurotransmitter metabolism by *Salvia officinalis* L. *BMC Complementary Medicine and Therapies*.

[59] Prajapati R., Park S. E., Seong S. H. (2021). Monoamine oxidase inhibition by major tanshinones from *Salvia miltiorrhiza* and selective muscarinic acetylcholine M4 receptor antagonism by Tanshinone I. *Biomolecules*.

[60] Hasselmo M. E. (2006). The role of acetylcholine in learning and memory. *Current Opinion in Neurobiology*.

[61] Farrell S. M., Tunbridge E. M., Braeutigam S., Harrison P. J. (2012). COMT Val158Met genotype determines the direction of cognitive effects produced by catechol-O-methyltransferase inhibition. *Biological Psychiatry*.

[62] Schacht J. P. (2016). *COMT* val158met moderation of dopaminergic drug effects on cognitive function: a critical review. *The Pharmacogenomics Journal*.

[63] National Research Council (US) Committee for the Update of the Guide for the Care and Use of Laboratory Animals (2011). *Guide for the care and use of laboratory animals*.

